# Remimazolam for General Anesthesia in a Patient with Severe Aortic Stenosis Undergoing High-Risk Surgery: A Case Report

**DOI:** 10.3390/medicina58101372

**Published:** 2022-09-29

**Authors:** Bo Rim Kim, Moo Soo Kim, Jae Hak Lee, Do Yeop Lee, Hye-bin Kim, Seok Kyeong Oh, Heezoo Kim

**Affiliations:** Department of Anesthesiology and Pain Medicine, Korea University Guro Hospital, Korea University College of Medicine, Seoul 08308, Korea

**Keywords:** aortic valve stenosis, case report, general anesthesia, hemodynamic monitoring, remimazolam

## Abstract

High-risk surgeries for patients with severe aortic stenosis (AS) are challenging for anesthesiologists and can result in hemodynamic deterioration and even mortality. We describe a case in which remimazolam was used to induce and maintain general anesthesia for a high-risk, noncardiac surgery accompanied by ongoing bleeding. An 86-year-old man with severe AS was scheduled to undergo proximal gastrectomy due to ongoing gastrointestinal bleeding and severe anemia. Remimazolam, a novel, ultra-short-acting benzodiazepine, was administered along with remifentanil for the induction and maintenance of general anesthesia. Throughout the anesthetic process, the patient’s cardiac index and systemic vascular resistance were well preserved without any vasopressor support. Remimazolam seems to have possible effectiveness as a relatively safe agent for the induction and maintenance of general anesthesia in patients with severe AS who are undergoing high-risk, noncardiac surgery with bleeding.

## 1. Introduction

Severe aortic stenosis (AS), one of the most challenging cases for anesthesiologists, can result in a fatal deterioration of cardiac output [1]. Anesthetic management in these patients is more difficult in cases of high-risk surgeries accompanied by bleeding. However, propofol, the most popular anesthetic agent, can cause significant myocardial depression and vasodilation in hemodynamically compromised patients; thus, its use requires extreme caution [2]. Etomidate and ketamine can be used for critically ill patients in terms of preserving systemic blood pressure; however, they have well-known adverse effects, such as adrenal toxicity, or tachycardia and hallucinations [3,4].

Remimazolam is a novel, ultra-short-acting benzodiazepine that has recently become available for general anesthesia purposes in Korea. Carrying the advantage of providing hemodynamic stability as a benzodiazepine, it can be used to maintain general anesthesia due to its short context-sensitive half-life and quick recovery time [5]. There have been only a few clinical reports of the use of remimazolam for general anesthesia in patients with severe AS [6,7]. We describe a case in which remimazolam was used for both induction and maintenance of general anesthesia in an elderly man with severe AS who was undergoing a high-risk, noncardiac surgery.

## 2. Case Presentation Section

Written informed consent was obtained from the patient for the publication of this case report.

We report the case of an 86-year-old man (height, 161.2 cm; weight, 55.2 kg) with severe AS who was scheduled to undergo proximal gastrectomy due to ongoing gastrointestinal bleeding accompanied by severe anemia. Without any transfusion, his hemoglobin level was less than 6.0 g/dL, and his lowest preoperative hemoglobin level was 3.9 g/dL. Endoscopy revealed several gastric angiodysplasias that required urgent surgery and could not be effectively controlled with argon plasma coagulation. Preoperative transthoracic echocardiography revealed a critically stenotic aortic valve with a peak/mean pressure gradient of 109.9/68.0 mmHg, a calculated aortic valve area of 0.87 cm^2^, a peak blood flow velocity of 5.16 m/s, and a concomitant concentric left ventricular hypertrophy. The left ventricular systolic function was preserved (ejection fraction, 60–65%) without relaxation abnormalities, and electrocardiography revealed normal sinus rhythm. The patient complained of dyspnea on exertion (New York Heart Association class II-III) and poor functional capacity, with a value of less than four metabolic equivalents. Preoperative chest radiography suggested pulmonary congestion, pleural effusion, and mild cardiomegaly. In addition, he had several underlying medical conditions, such as chronic obstructive pulmonary disease, chronic kidney disease (glomerular filtration rate, 41.9 mL/min/1.73 m^2^), and a history of an intracranial abscess.

The initial plan for laparoscopic surgery was changed to laparotomy after sufficient discussion with the surgeon, as a decrease in preload could have been devastating for this patient. After consultations with the cardiology and cardiac surgery departments, which revealed the patient’s risks, benefits, and prognosis, a decision to conduct surgery was made. An extracorporeal membrane oxygenation device was prepared in case of emergency.

After the patient was admitted to the operating room, pulse oximetry, non-invasive blood pressure, and electrocardiography were monitored along with patient state index (PSi) with near-infrared spectroscopy-based cerebral oximetry (SedLine^®^ with O3^®^ Regional Oximetry, Masimo Corporation, Irvine, CA, USA). Prior to general anesthesia induction, an arterial catheter was inserted into the radial artery and connected to the arterial waveform analysis system (Flotrac: Edwards Lifesciences, Irvine, CA, USA) for continuous arterial pressure with cardiac output monitoring and blood sampling. To prevent postoperative nausea and vomiting, palonosetron (0.075 mg) and dexamethasone (5 mg) were administered preoperatively as a prophylactic treatment.

**Figure 1:**
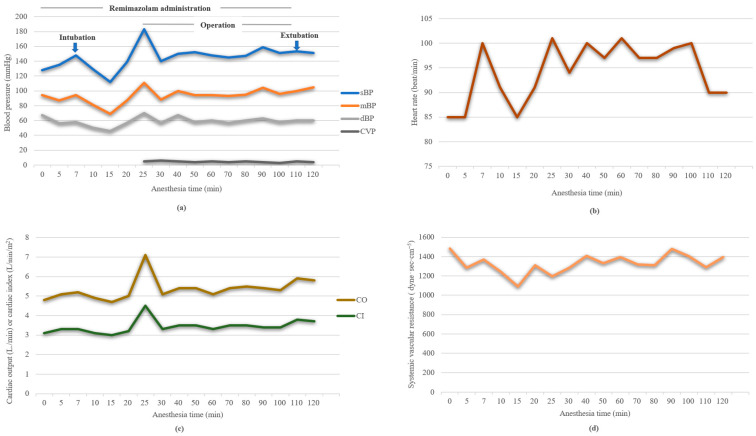
Hemodynamic changes during the surgery. sBP, systolic blood pressure; mBP, mean blood pressure; dBP, diastolic blood pressure; CVP, central venous pressure; CO, cardiac output; CI, cardiac index. (**a**) Changes in blood pressure during the surgery. (**b**) Changes in heart rate changes during the surgery. (**c**) Changes in cardiac output and cardiac index during the surgery. (**d**) Changes in systemic vascular resistance during the surgery.

General anesthesia was induced with remimazolam (6 mg/kg/h) and a target-controlled infusion of remifentanil (target effect-site concentration [Ce], 3 ng/mL) was administered. After 106 s, confirming the patient’s loss of consciousness, 70 mg of rocuronium was administered at an adjusted infusion rate of 1 mg/kg/h of remimazolam. Anesthesia induction and endotracheal intubation were successfully performed without hemodynamic disturbance (Figure 1). The PSi value at the time of the patient’s loss of consciousness was 72 and continued to decrease to 45 at the time of intubation. Ultrasound-guided central venous catheter insertion into the right internal jugular vein was performed, and norepinephrine and vasopressin were prepared in case of hemodynamic deterioration.

During surgery, anesthesia was maintained with remimazolam (1.0–2.0 mg/kg/h) and remifentanil (Ce, 0.5–3 ng/mL), keeping the PSi value in the range of 45–53. The patient was ventilated with a tidal volume of 8 mL/kg of ideal body weight, a respiratory rate of 10–13/min targeting PaCO_2_ of 35–45 mmHg, and a positive end-expiratory pressure of 5 cmH_2_O. Intraoperative hemodynamic changes are shown in Figure 1. Throughout the perioperative period, the mean arterial pressure was maintained above 65 mmHg without the requirement of vasopressors or inotropes. Cardiac index and systemic vascular resistance were preserved at 3.0–4.5 L/min/m^2^ and 1089–1483 dyne⋅sec⋅cm^−5^, respectively. In terms of cerebral oximetry, the baseline values measured at room air were 57/58 (left/right), and the intraoperative values ranged from 59 to 62. Considering his status of ongoing bleeding and his hemoglobin level of 8.9 g/dL, he continued to receive transfusions during the operation.

At the end of the surgery, the patient received a bolus of fentanyl (50 µg) and paracetamol (1000 mg) infusion along with intravenous patient-controlled analgesia. Remimazolam was discontinued, whereas remifentanil was maintained at 1 ng/mL for smooth emergence. Sugammadex (2 mg/kg) was administered after train-of-four count monitoring for prompt neuromuscular reversal. The patient regained consciousness 7 min after remimazolam cessation and was extubated 1 min later, with sufficient spontaneous breathing. The PSi values were 82 and 97 at the time of extubation and discharge from the operating room, respectively. The overall operative time was 81 min, and the anesthesia time was 120 min. Two units of red blood cells and fresh frozen plasma were administered in addition to 750 mL of a crystalloid, and the estimated blood loss and urine output were 200 mL and 80 mL, respectively. The patient was transferred to the intensive care unit for one day of close monitoring. In his kidney and liver function tests, no significant change was detected during the perioperative period (Table 1). He was hemodynamically stable and was discharged from the hospital on postoperative day 8.

## 3. Discussion

It is important to select the optimal anesthetic agents for critically ill patients. Remimazolam has been shown to effectively maintain hemodynamic stability in American Society of Anesthesiologists (ASA) class III patients undergoing elective noncardiac surgery, but the evidence for its use in patients with critical cardiac disease undergoing noncardiac surgery is lacking [8].

To the best of our knowledge, there have been few clinical reports of remimazolam-based general anesthesia in patients with AS [6,7,9]. Furuta et al. [6] reported a case of successful general anesthesia with remimazolam for total mastectomy in an old woman with severe AS. However, it was difficult to evaluate the hemodynamic effect of remimazolam in this case since noradrenaline was used from the beginning of induction to maintain vascular tone. In contrast, general anesthesia in our case was induced and maintained with a combination of remimazolam and remifentanil without any vasoconstrictor support. In addition, the patient underwent proximal gastrectomy after urgent admission, which is considered a high-risk surgery due to its associated mortality rate of 14.8% in patients aged 65 years or older [10]. Due to ongoing bleeding accompanied by severe anemia, our patient was considered to be at a higher risk than others who underwent conventional proximal gastrectomy. We demonstrated that remimazolam was a safe, alternative anesthetic agent in this high-risk case. Other recent studies have reported that remimazolam induces general anesthesia without hemodynamic instability in patients undergoing transcatheter aortic valve replacement or surgical aortic/mitral valve replacement [7,9]. In these studies, either sevoflurane or propofol with dexmedetomidine was used to maintain anesthesia when the patient was confirmed to be unconscious. This was different from our case, where remimazolam was used for the entire anesthetic process in noncardiac surgery.

Benzodiazepines mainly act on the gamma-aminobutyric acid-A receptor. Due to their minimal cardiovascular effect, benzodiazepines have been considered relatively safe induction agents for elderly or compromised patients [11]. However, they are not suitable for continuous infusion or maintenance of general anesthesia because of their prolonged elimination half-life and their associated accumulation of active metabolites [12]. For maintenance, additional inhalational or intravenous agents should be used, which usually require delicate titration in terms of vasodilation and myocardial suppression. However, remimazolam has a unique metabolism that involves rapid hydrolysis by plasma esterase-producing inactive metabolites, allowing it to be used for maintenance purposes [5]. In addition, remimazolam has been shown to cause relatively less hemodynamic deterioration even in high-risk patients with ASA-III or hepatorenal insufficiency [8,13]. In accordance with the previous reports, general anesthesia was performed without hemodynamic deterioration or emergence delay despite chronic kidney disease in our patient. Furthermore, his hepatorenal function was not adversely affected compared to preoperative laboratory tests (Table 1).

Our patient presented with ongoing bleeding, anemia, and severe AS. Patients with severe AS are very preload-dependent; thus, maintaining adequate preload is the main goal of anesthetic management in these patients. In this case, the preload was expected to already be lowered due to hemorrhage; hence, anesthetic-induced critical preload reduction could have occurred owing to a reduction in vascular tone and increased venous pooling. However, remimazolam would have preserved the preload by reducing venous pooling and maintaining systemic vascular resistance and contractility, contributing to hemodynamic stability. The patient was managed with a well-preserved cardiac index under anesthesia. In terms of cardiovascular stability in high-risk cases with bleeding, remimazolam appears to be a relatively safe choice.

A previous study reported that electroencephalogram-based anesthetic depth monitoring devices such as bispectral index and PSi showed relatively high values during anesthesia with remimazolam [14]. In our case, the PSi was used to assess the patient’s depth of anesthesia. During induction of general anesthesia, PSi seemed slightly delayed to reflect the decline in consciousness level, which is consistent with a previous report on the pharmacodynamics of remimazolam [15]. Remimazolam was subsequently infused at 1–2 mg/kg/h during anesthesia maintenance, keeping the PSi within the adequate range (45–53) without signs of awakening. The PSi values reflected the recovery of consciousness with acceptable accuracy during the emergence process. There was no recall or awareness of intraoperative events during the postoperative period.

At the end of the surgery, remimazolam infusion was discontinued, with prompt neuromuscular recovery using sugammadex. Although the effect of remimazolam could have been reversed by flumazenil, we planned a spontaneous recovery with concerns regarding the rapid increase in vascular resistance and the possibility of re-sedation. To facilitate spontaneous recovery, remimazolam was carefully titrated throughout the surgery to maintain an optimal anesthetic depth. In addition, analgesic agents were administered before and during emergence to prevent a sudden increase in sympathetic tone due to postoperative pain. Consequently, the patient regained consciousness and was extubated in a stable condition without an antagonist. During hospitalization, no adverse effects of remimazolam, such as headache, nausea, fever, or withdrawal symptoms, were reported.

In this high-risk case, remimazolam with remifentanil was used to induce and maintain general anesthesia safely and effectively. Cardiac output and systemic vascular resistance were stable throughout the anesthetic procedure without vasopressor support. In conclusion, remimazolam seems to have the possibility to be a safe and effective option for general anesthesia in patients with severe AS who are undergoing high-risk, noncardiac surgery with bleeding. However, the usefulness of remimazolam in critically ill patients cannot be conclusive from this report, as this report was limited to a single case. Well-designed, comparative investigations are still required to establish remimazolam as a competitive agent for high-risk cases.

## Figures and Tables

**Table 1:** Perioperative kidney and liver function tests.

	Laboratory Tests	Preoperative	Postoperative
Kidney function tests	Blood urea nitrogen (mg/dL)	39.2	28.7
	Serum creatinine (mg/dL)	1.49	1.46
Liver function tests	AST (unit/L)	18	30
	ALT (unit/L)	8	20
	Alkaline phosphatase (unit/L)	41	62
	Total bilirubin (mg/dL)	0.83	0.86
	Albumin (g/dL)	3.2	3.1

AST, Aspartate aminotransferase; ALT, Alanine aminotransferase.

## Data Availability

Not applicable.
